# Gene Duplication in a Patient With Usher Syndrome Type 2C: A Case Report

**DOI:** 10.7759/cureus.83212

**Published:** 2025-04-29

**Authors:** Sebastián J Ruiz Matos, Krytsia A Negrón Lugo, Natalio Izquierdo

**Affiliations:** 1 Department of Ophthalmology, University of Puerto Rico, Medical Sciences Campus, San Juan, PRI; 2 Department of Ophthalmology, Universidad Central del Caribe, School of Medicine, Bayamón, PRI

**Keywords:** adgrv1, congenital hearing loss, retinitis pigmentosa, ush2c, usher syndrome

## Abstract

Variants of the *ADGRV1* gene cause Usher syndrome type 2C (USH2C). There are three types of Usher syndrome, all inherited in an autosomal recessive pattern. USH2C classically causes sensorineural hearing loss and progressive retinitis pigmentosa (RP) occurring in adolescence or adulthood. In this study, we report the case of a 34-year-old male with congenital sensorineural and progressive hearing loss and peripheral vision loss with a fundus examination of pale optic nerve with a cup-to-disk ratio of 0.40, arteriolar attenuation, arteriovenous ratio 2/5 with choroid-retinal degeneration, peripheral bony spicules, and fundus tessellation. Visual field test (30-2 Carl Zeiss Meditec, Inc.) showed that the patient had a mean deviation of -21.22 dB (p < 0.5) and 7.51 dB (p < 0.5) in the right and left eye, respectively. Upon full-field electroretinogram (ERG), the patient had non-recordable photopic and scotopic ERG responses bilaterally. The patient’s macular optical coherence tomography showed an average thickness of 221 µm and a total macular volume of 8 mm^3 ^in the right eye, and an average thickness of 215 µm and a total macular volume of 7.8 mm^3 ^in the left eye. Based on these ocular findings, the patient was clinically diagnosed with RP. Genetic testing with exome sequencing showed a homozygous pathogenic copy number variation (4) at exons 79-84 of the *ADGRV1 *gene. Our case highlights the importance of recognizing the specific type of variant affecting a gene in hereditary diseases such as RP, as disease severity may be influenced by the nature of the variant.

## Introduction

Usher syndrome (USH) is the most common disease that affects both the visual and auditory systems simultaneously. Patients usually have sensorineural hearing loss and retinitis pigmentosa (RP). Some of the patients, for example, those with USH type 1 (USH1), can present with vestibular impairment as well [1]. Symptoms of RP include nyctalopia and a loss of peripheral vision through the progressive degeneration of retinal cells. The prevalence of the syndrome is 3-6.2 out of 100,000 people [2].

Three types of Usher syndrome have been described, all of which are inherited as an autosomal recessive trait. The various types of the syndrome differ according to the onset and severity: USH1 leads to profound congenital hearing loss and early childhood vision loss; USH type 2 (USH2) leads to congenital hearing loss but vision loss usually begins in adolescence or adulthood; and USH type 3 (USH3) involves late-onset hearing and late-onset vision loss [3].

Nine genes have been associated with USH. The most common variant leading to USH1 occurs in the *MYO7A* gene. It encodes for Myosin 7A, which is fundamental for the development and maintenance of stereocilia. These stereocilia are present both in the cochlea and the retinal pigment epithelium (RPE) [3]. Similarly, other genes such as *CDH23* and *USH2A* encode for proteins found in the cochlea and retina [3,4]. Genetic variants in the *USH2A* gene most commonly lead to USH2 [3]. About 9% of patients with USH2 have variants in the *ADGRV1* gene [5]. *ADGRV1* is the fourth most common gene in USH and encodes for a very large G protein-coupled receptor-1 (VLGR1), which is the largest surface receptor protein and is expressed in the developing central nervous system [5,6]. VLGR1 functions as a fibrous membrane linkage in cilia of inner-ear hair cells and photoreceptors [5].

We report the case of a patient with a pathogenic copy number variation (4) at exons 79-84 of the* ADGRV1* gene who had RP and sensorineural hearing loss as part of the USH type 2C (USH2C).

## Case presentation

A 34-year-old male patient was referred to the Ophthalmic Genetics Clinic in San Juan, Puerto Rico, due to moderate bilateral congenital and progressive sensorineural hearing loss since birth, nyctalopia, and progressive vision loss in both eyes. These symptoms had worsened during the last five years, deteriorating his quality of life. The patient denied using ocular medications, as well as any history of ocular surgery or trauma. There was a history of consanguinity, and both parents shared the same last name.

The patient underwent a comprehensive ophthalmic evaluation by at least one of the authors (NJI), visual field testing, a full field electroretinogram test (ERG), optical coherence tomography (OCT), and a gene sequencing and deletion/duplication analysis using next-generation sequencing (NGS) (Invitae Corporation, San Francisco, CA, USA).

His best-corrected visual acuity was 20/25 and 20/20 in the right and left eye, respectively. Refraction was -4.75 +2.25 x 110 and -4.75 +2.50 x 95 in the right and left eye, respectively. On fundus examination, the patient had a pale optic nerve with a cup-to-disk ratio of 0.40, arteriolar attenuation, arteriovenous ratio 2/5 with choroid-retinal degeneration, peripheral bony spicules, and fundus tessellation.

**Figure 1:**
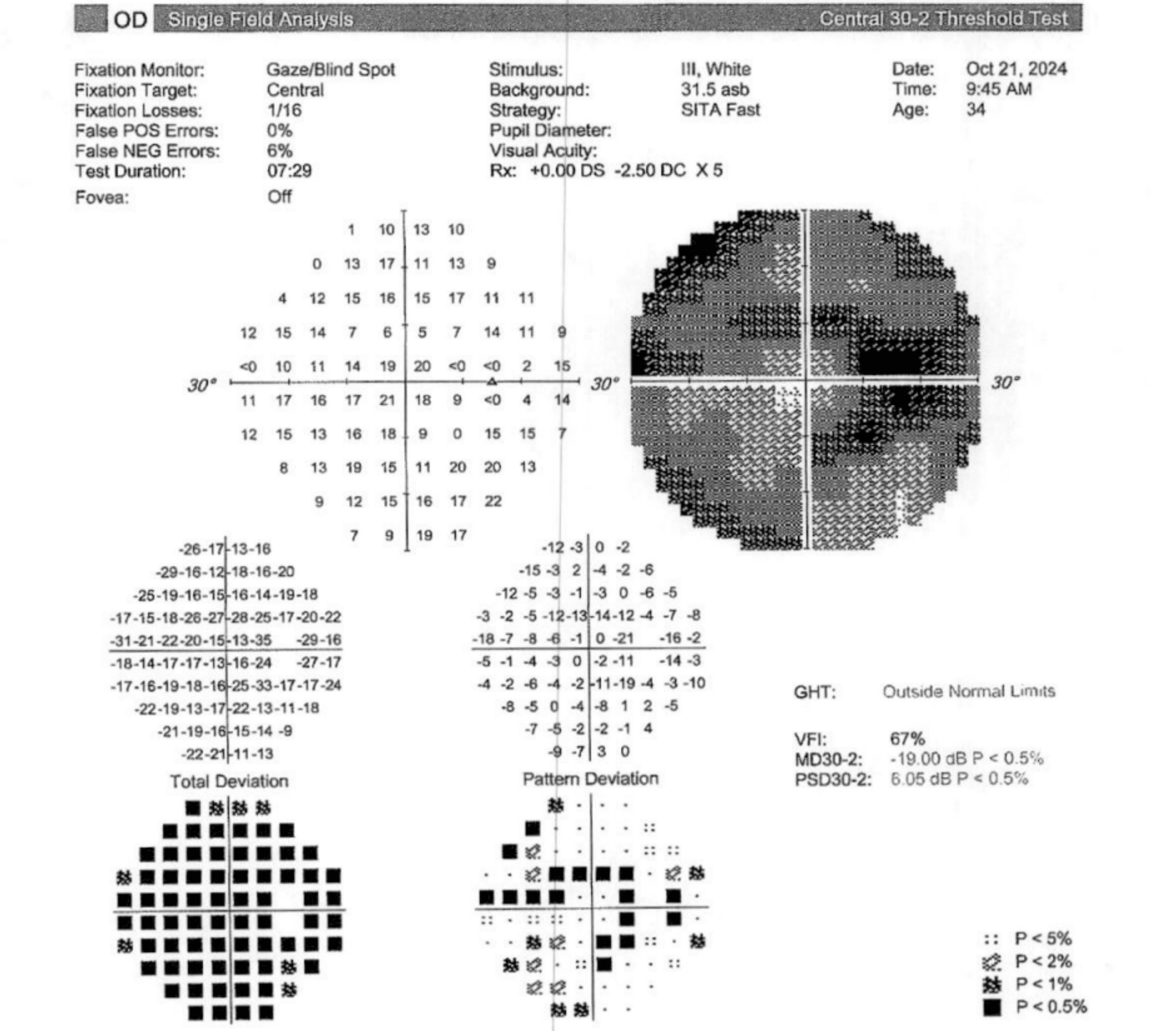
Visual field testing (30-2 Carl Zeiss Meditec, Inc.) shows a significantly decreased mean deviation (p < 0.5) in the right eye. GHT: glaucoma hemifield test; VFI: visual field index; MD: mean deviation; PSD: pattern standard deviation

**Figure 2:**
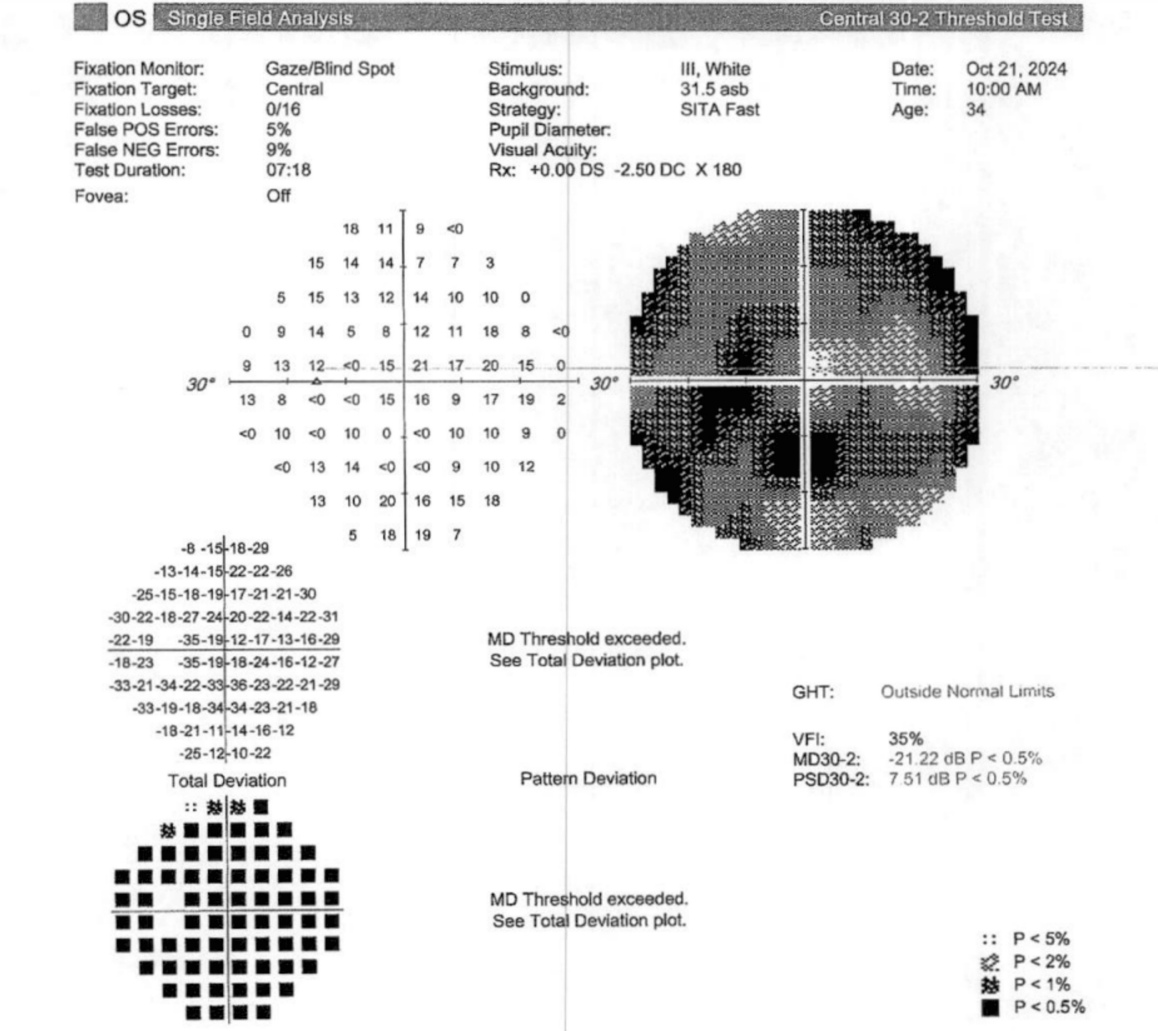
Visual field testing (30-2 Carl Zeiss Meditec, Inc.) shows a significantly decreased mean deviation (p < 0.5) in the left eye. GHT: glaucoma hemifield test; VFI: visual field index; MD: mean deviation; PSD: pattern standard deviation

Visual field testing (30-2 Carl Zeiss Meditec, Inc.) showed a mean deviation of -19.00 dB (p < 0.5) and a pattern deviation of 6.05 dB (p < 0.5) in the right eye and a mean deviation of -21.22 (p < 0.5) and a pattern deviation of 7.51 dB (p < 0.5) in the left eye, as depicted in Figure 1 and Figure 2, respectively.

**Figure 3:**
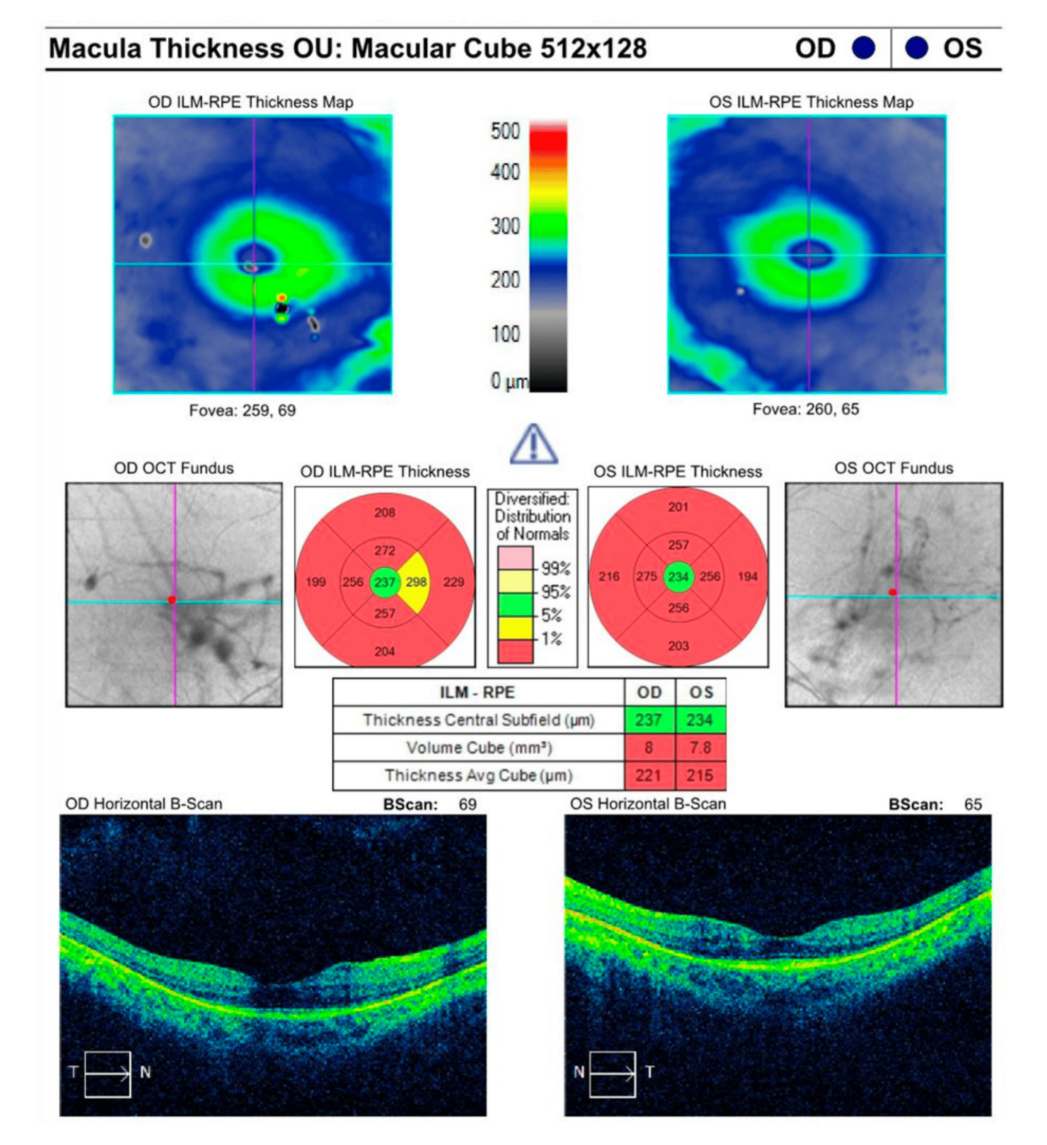
Macular optical coherence tomography study shows decreased macular thickness and volume in both eyes. OCT: optical coherence tomography; RPE: retinal pigment epithelium; ILM: inner limiting membrane

Upon full-field ERG, the patient had non-recordable photopic and scotopic ERG responses bilaterally. In RP, photoreceptor cell degeneration and disease progression can present as a decrease in macula thickness and volume. As depicted in Figure 3, the patient’s macular OCT showed an average thickness of 221 µm (normal range: 257.1-295 µm) and a total macular volume of 8 mm^3^ (normal range: 9.39-10.75 mm^3^) in the right eye and an average thickness of 215 µm and a total macular volume of 7.8 mm^3^ in the left eye.

A clinical diagnosis of RP was reached. A saliva sample was sent for genetic testing. Gene exome sequencing and deletion/duplication analysis using NGS (Invitae Corporation, San Francisco, CA, USA) was positive for a homozygous pathogenic (gain) copy number variation (4) at exons 79-84 of the *ADGRV1 *gene located in the 5q14 region on chromosome 5.

The patient was educated about the increased risk of inherited autosomal recessive diseases associated with consanguinity and was also informed about the potential genetic risks for relatives. The patient was referred to a geneticist.

## Discussion

Patients with USH have a combination of sensorineural hearing loss and retinal dystrophy. The syndrome is divided into three major clinical subtypes, with USH2 being the most prevalent [7]. USH2 is characterized by congenital moderate to severe but relatively stable sensorineural hearing loss, normal vestibular function, and late-onset RP, which typically begins at a young adult age [5]. Our patient had a history of moderate congenital bilateral sensorineural hearing loss since birth and was recently diagnosed with progressive, painless peripheral vision loss due to RP. The patient did not have vestibular impairment as part of the clinical presentation of USH2 phenotypes.

Patients with USH2C usually have biallelic, homozygous, or compound heterozygous variants in the *ADGRV1* gene [8,9]. These often involve pathogenic loss-of-function variants, such as nonsense or frameshift variants, resulting in biallelic null alleles and thus complete loss of protein function [5]. Our patient had pathogenic duplication of exons 79-84 in the *ADGRV1* gene, which introduces a distinct pathogenic mechanism. In contrast to the aforementioned variants, duplication likely disrupts the balance of protein function by altering its structure, splicing, or expression levels, which can interfere with the Usherin protein network critical for sensory cilia [10]. Both mechanisms ultimately impair *ADGRV1* function; however, duplication reflects a novel molecular pathway of dysfunction.

Copy number variations (CNVs) play a significant role in the pathogenicity of inherited retinal disorders (IRDs). According to a study analyzing 500 patients with NGS, 44 (8.8%) patients with IRD had CNVs [11]. Researchers found that only 5 out of 44 patients with pathogenic CNVs carried causal duplications. They found CNVs in 18 genes, with only one tandem duplication found in the *ADGRV1* gene [11]. In our patient, a targeted gene panel analysis using NGS identified a loss-of-function pathogenic variant in the *ADGRV1 *gene, characterized by an increased CNV of 4 in exons 79-84. Similar CNVs have been found in patients with USH [2,12]. This finding underscores that, while CNVs are critical contributors to unsolved IRD cases, duplications are relatively rare compared to more commonly identified loss-of-function variants [8,11].

USH2C and USH2A are both characterized by progressive retinal degeneration and hearing loss but differ in their genetic basis and disease severity. USH2C is caused by variants in the *ADGRV1* gene, encoding VLGR1, a large G-protein-coupled receptor involved in cellular signaling [6]. At the same time, USH2A results from variants in the *USH2A* gene, which encodes Usherin, a protein critical for cell adhesion in the retina and cochlea. Phenotypically, USH2C presents with severe and widespread retinal dysfunction, with no regions of normal rod function, whereas USH2A shows variable severity, with some patients retaining regions of normal retinal function [2]. Both genes play essential roles in sensory cell structure and function, making them critical targets for gene therapy and pharmacological interventions to address the shared and distinct pathophysiological mechanisms of these subtypes in the future [5].

## Conclusions

Our case highlights the importance of recognizing the specific type of variant affecting a gene in hereditary diseases such as RP, as disease severity may be influenced by the nature of the variant. This is among the first reported cases of USH2C with a pathogenic variant in the *ADGRV1* gene due to multiple (4) exon duplications. Further genetic studies in patients with syndromic RP are warranted.

## References

[1] Schwartz SB, Aleman TS, Cideciyan AV (2005). Disease expression in Usher syndrome caused by VLGR1 gene mutation (USH2C) and comparison with USH2A phenotype. Invest Ophthalmol Vis Sci.

[2] Aparisi MJ, Aller E, Fuster-García C (2014). Targeted next generation sequencing for molecular diagnosis of Usher syndrome. Orphanet J Rare Dis.

[3] Keats BJ, Corey DP (1999). The usher syndromes. Am J Med Genet.

[4] Bolz H, von Brederlow B, Ramírez A (2001). Mutation of CDH23, encoding a new member of the cadherin gene family, causes Usher syndrome type 1D. Nat Genet.

[5] Daich Varela M, Wong SW, Kiray G (2023). Detailed clinical, ophthalmic, and genetic characterization of ADGRV1-associated Usher syndrome. Am J Ophthalmol.

[6] McMillan DR, Kayes-Wandover KM, Richardson JA, White PC (2002). Very large G protein-coupled receptor-1, the largest known cell surface protein, is highly expressed in the developing central nervous system. J Biol Chem.

[7] Rosenberg T, Haim M, Hauch AM, Parving A (1997). The prevalence of Usher syndrome and other retinal dystrophy-hearing impairment associations. Clin Genet.

[8] Wei C, Yang L, Cheng J (2018). A novel homozygous variant of GPR98 causes usher syndrome type IIC in a consanguineous Chinese family by next generation sequencing. BMC Med Genet.

[9] Zhang N, Wang J, Liu S, Liu M, Jiang F (2018). Identification of two novel compound heterozygous mutations of ADGRV1 in a Chinese family with Usher syndrome type IIC. Ophthalmic Genet.

[10] Rice AM, McLysaght A (2017). Dosage-sensitive genes in evolution and disease. BMC Biol.

[11] Zampaglione E, Kinde B, Place EM (2020). Copy-number variation contributes 9% of pathogenicity in the inherited retinal degenerations. Genet Med.

[12] Besnard T, Vaché C, Baux D (2012). Non-USH2A mutations in USH2 patients. Hum Mutat.

